# Development and pilot testing of a mental healthcare plan in Nepal

**DOI:** 10.1192/bjp.bp.114.153718

**Published:** 2016-01

**Authors:** M. J. D. Jordans, N. P. Luitel, P. Pokhrel, V. Patel

**Affiliations:** **M. J. D. Jordans**, PhD, Research and Development Department, HealthNet TPO, Amsterdam, The Netherlands and Center for Global Mental Health, Institute of Psychiatry, Psychology and Neuroscience, King's College London, London, UK; **N. P. Luitel**, MA, **P. Pokhrel**, BA, Research Department, Transcultural Psychosocial Organization (TPO), Kathmandu, Nepal; **V. Patel**, MRCPsych, PhD, Centre for Global Mental Health, London School of Hygiene and Tropical Medicine, London and Sangath Centre, Goa, India

## Abstract

**Background**

Mental health service delivery models that are grounded in the local context are needed to address the substantial treatment gap in low- and middle-income countries.

**Aims**

To present the development, and content, of a mental healthcare plan (MHCP) in Nepal and assess initial feasibility.

**Method**

A mixed methods formative study was conducted. Routine monitoring and evaluation data, including client flow and reports of satisfaction, were obtained from patients (*n* = 135) during the pilot-testing phase in two health facilities.

**Results**

The resulting MHCP consists of 12 packages, divided over community, health facility and organisation platforms. Service implementation data support the real-life applicability of the MHCP, with reasonable treatment uptake. Key barriers were identified and addressed, namely dissatisfaction with privacy, perceived burden among health workers and high drop-out rates.

**Conclusions**

The MHCP follows a collaborative care model encompassing community and primary healthcare interventions.

In recent years there have been many appeals for increased and improved mental health services globally,^1,2^ especially in low- and middle-income countries (LMIC) where such care is unavailable to most people.^3^ The World Health Organization (WHO) advocates the integration of mental health into primary healthcare, as a strategy to overcome the enormous gap between people in need of treatment and the availability of such care, and has developed guidelines for the treatment of mental health, neurological and substance use disorders in primary care.^4^ Little is known about how to transfer such guidelines into an actual service delivery framework that is feasible, sustainable and effective in practice in LMIC. Nepal is one of many countries where mental healthcare is very scarce and entirely limited to urban centres and hospitals. The lack of human and financial resources to establish mental healthcare is worsened by the fragility of the health system as a result of a decade-long armed conflict and political instability dating from the peace treaty signed in 2006. A recent situation analysis confirms the bleak situation of mental healthcare, yet also demonstrates some tentative improvements in recent years.^5^ A policy framework for the integration of mental health into primary healthcare exists in Nepal. Although mostly dormant, the policy does provide opportunities to build up and shape the development of a mental healthcare strategy that adequately responds to the existing needs and barriers, something that is similar in other humanitarian settings.^6^ Furthermore, there are a number of prior initiatives towards mental healthcare development in Nepal that can be built upon.^7–9^

The PRogramme for Improving Mental health carE (PRIME, www.prime.uct.ac.za) aims to develop and evaluate a strategy for the integration of mental health into primary healthcare, specifically for depression, psychoses and alcohol use disorder.^10^ The programme is operating in districts in five LMIC (Ethiopia, India, Nepal, South Africa and Uganda). In Nepal it is implemented by the non-governmental organisation (NGO) Transcultural Psychosocial Organization (TPO) Nepal, which works throughout Nepal on implementing and evaluating mental healthcare. In this paper we present the mental healthcare plan (MHCP) that has been developed in collaboration with the Ministry of Health and Population. We will summarise the steps of development, present its content and report results of the pilot testing of the MHCP and how this has led to a final plan ready for implementation and evaluation.

## Method

Nepal, with a total population of 28 million, has come out of a violent insurgency that took place between 1996 and 2006, initiated by the Communist Party of Nepal – Maoist and fuelled by poverty, unequal distribution of wealth, social marginalisation and disappointment with state governance.^11^ Since the 2006 peace agreement the country has been in a devastating political gridlock around the drafting of a new constitution. PRIME has been implemented in Chitwan, a district on the southern plains of Nepal (online Fig. DS1). Chitwan has a population of 575 058 people, 73% of whom live in a rural setting and a literacy rate of 70% (compared with the national average of 54%).^12^ With two psychiatrists and a psychiatric ward in the district public hospital, the district is better off compared with most in Nepal, but similarly to the rest of the country, it has no mental health services as part of the basic healthcare package delivered in locally available primary care.

The development of the MHCP consisted of two stages: (a) formative research informing the content and structure of the care system, followed by (b) pilot testing to adapt and fine-tune the plan. The formative study employed a mixed methods design. First, we engaged an expert panel (*n* = 26) in a structured exercise to prioritise the mental health problems to be targeted in the MHCP. Second, we organised workshops involving policy makers and service providers (*n* = 19) to develop a theory of change (ToC), which served as a roadmap of intermediate steps towards increased coverage of evidence-based mental health services.^13^ Third, we conducted in-depth interviews and focus-group discussions with key stakeholders (*n* = 117) to assess perceptions and barriers related to integrating mental health into primary healthcare (based on the already developed ToC). Detailed description of the formative study has been published previously.^14^

Following development of the first version of the MHCP, piloting was carried out in two health facilities (the only available ones in two villages, Meghauli and Divyanagar, serving a population of approximately 28 000). In terms of socio-demographic characteristics of the population, geography and access to healthcare, these locations were comparable with the part of the district where the MHCP will be implemented (12 health facilities). All nine health workers from both facilities received the designed training. Piloting started in April 2013 and continued until February 2014. The objectives were twofold: to pilot the implementation of the MHCP and identify challenges and barriers, and to assess patients and providers' perceptions of the MHCP. Parts of the MHCP that require a long running time or involve the health system at-large were excluded given the relatively brief period and small scale of the piloting.

We obtained routine monitoring data for all patients during the pilot phase (*n* = 135) and additionally administered evaluation questionnaires to a random selection of these patients (*n* = 45) as well as to service providers (*n* = 11). The questionnaire included items on level of satisfaction, degree of met expectations and degree of perceived changes. The questionnaire consisted of 11 structured items and 5 open-ended questions. Four items were adapted from the Client Satisfaction Questionnaire,^15^ and others were developed for the purpose of this study. Interviews were conducted in the health facility for the service providers and at home for the patients. All questionnaires were verbally administered by research assistants who read the questions out loud and recorded responses. This was done to control for the variable literacy aptitude of participants and because of respondents' unfamiliarity with completing questionnaires.

Descriptive analyses were run on the quantitative data, using SPSS 20.0. Furthermore, a group of patients (*n* = 28) who dropped out of care after only one contact were followed-up in order to understand the reasons why they dropped out. A framework analysis approach was utilised to examine the major themes that emerged from the collation of qualitative responses,^16^ coding was done by hand given the small data-set. The study conforms with the requirements of the Declaration of Helsinki^17^ and received ethical approval from the Nepal Health Research Council.

## Results

### Presentation of the MHCP

The MHCP targets psychoses, depression and alcohol use disorders. As a result of the high priority given to epilepsy by the expert panel during the formative study, this disorder was added. The MHCP that has been developed consists of 12 care packages divided over three levels (health organisation, health facility and community), compatible with the PRIME framework.^10^ The WHO Mental Health Gap Action Programme (mhGAP) intervention guide forms the core of the treatments provided at the health facility.^4^ The basic structure of the plan is further shaped by the outcomes of the formative study, primarily by the ToC that outlines the key building blocks of the package. Furthermore, it demonstrates that there is widespread endorsement of the aim to integrate mental health into community – and primary healthcare systems. A number of key challenges are identified (including the already burdened primary healthcare workers, stigma attached to mental health problems, insufficient mandate for healthcare staff to perform mental healthcare), leading to adaptations in the structure of the care package.^14^ Below we present an overview of the MHCP, including adaptations that are made as a result of the formative study (Tables 1 and 2).

**Table 1:** Mental healthcare plan matrix

Level	Awareness packages	Detection packages	Treatment packages	Recovery packages
1. Health organisation	1.1 Engagement and advocacy			

2a. Specialist mental health services	2.1 Referral for management of complex or treatment-resistant cases		

2b. Health facility (primary healthcare)	2.2 Service provider awareness raising and stigma reduction	2.3 Screening and assessment	2.4 Basic psychosocial support 2.5 Focused psychosocial support 2.6 Pharmacological treatment	2.7 Continuing care

3. Community	3.1 Mass sensitisation and stigma reduction	3.2 Community informant case detection (CIDT)	3.3 Advanced psychosocial support	3.4 User group mobilisation

CIDT, community informant detection tool.

**Table 2:** Overview training and supervision

		Human resource allocation estimates^a^	Training		Supervision	
Level and package	Service provider		Type	Duration	Frequency	Supervisor
Health facility						
Awareness raising and stigma reduction	All facility personnel	–^b^	Introduction (level 1)^c^	2 days	n/a	n/a
Basic psychosocial support	Healthcare providers^d^	–^b^	Support skills (level 2)^c^	2 days	Monthly	Community counsellor
Assessment	Prescribers	2.4 FTE/100 000 (for 0.08 FTE per provider)	mhGAP (level 3a)^c^	5 days	Monthly case conference	Psychiatrist
Pharmacological treatment					Once every 2 months managerial	District public health office
Focused psychosocial support	Non-prescribers	2.4 FTE/100 000 (for 0.08 FTE per provider)	Brief psychological treatments (level 3b)^c^	5 days	Monthly group supervision	Community counsellor

Community						
Community case detection	Targeted community members^e^	4.5 FTE/100 000 (for 0.05 FTE per provider)	CIDT and community mobilisation	2 days	Monthly group monitoring	Community counsellor
Mass sensitisation and stigma reduction				1 day		
Advanced psychosocial support	Community counsellors	5.0 FTE/100 000 (for 1.0 FTE per provider)	Course for generic counselling skills Protocolised psychological treatments	5 months 20 days	Once per month	Psychologist
User group mobilisation	Service users	N/A	Peer support group formation	5 days	Ongoing contact	Community counsellor

FTE, full-time equivalent; mhGAP, World Health Organization (WHO) Mental Health Gap Action Programme; CIDT, community informant detection tool ; N/A, not applicable.

a. During the PRogramme for Improving Mental health carE (PRIME) implementation phase, 81 health workers and 6 counsellors will be involved in service delivery for a catchment population of approximately 130000.

b. Included in calculations below.

c. The levels refer to the accumulating training structure, wherein all health facility personnel receive level 1, all healthcare providers an additional level 2, and some health workers an additional level 3.

d. Prescribers and non-prescribers combined.

e. Female community health volunteers and mothers group members.

### Healthcare organisation platform

A single care package is planned for the healthcare organisation platform: engagement and advocacy (package 1.1 in Table 1). Presently, the level of awareness about mental health is very low among health managers and policy makers, both at the national and the district level. Yet their understanding and engagement is vital for the ultimate sustainability of the MHCP. The objective of this package is to sensitise leaders about the need for mental healthcare and develop support within the health system for such integrated care (including issues such as drug supply chain management and health management information systems). As part of this package, regular workshops with relevant divisions in the government system are organised. So far this has resulted in commitment from the Ministry of Health and Population for the procurement and supply of psychotropic drugs, and providing time for healthcare staff for training and mental healthcare delivery, for the duration of the programme (5 years, 2013–2018).

### Health facility delivery platform

A central objective of the MHCP is to deliver non-stigmatising care by competent health workers, to improve the social, economic and health outcomes of people with mental disorders. A number of care packages are included to achieve this, all corresponding to different levels of training (Table 2 and online Fig. DS2).

First, service provider awareness and stigma reduction (package 2.2 in Table 1). The objective of this package is to increase knowledge about mental health problems and services among all health facility staff, to change the perception towards mental health and reduce mental health stigma. A 2-day training course for all staff covers basic information on mental health problems, causes and treatment, as well as common misconceptions about mental health. In addition to the knowledge-driven training course, a targeted stigma intervention consists of organising interactive workshops bringing primary healthcare workers, patients and their family members together to discuss attitudes towards mental health. This social-contact intervention targeting primary healthcare workers aims to teach basic skills to reduce stigma, fear of violence, fear of contagion associated with provision of mental healthcare and develop a locally specific action plan for countering stigma within the health facility.

Second, clinical staff will be trained in the assessment and management of priority mental disorders, including pharmacological and psychosocial support components, following the mhGAP intervention guide. All primary healthcare workers are trained to provide basic psychosocial support to people with mental health problems and their families (package 2.4 in Table 1). Practically, the package comprises psychoeducation to help patients and their caregivers better understand the problems and treatment, emotional support through empathetic engagement to reduce distress and case management for practical support. One of the key challenges raised by the formative research is the risk of overburdening the primary healthcare workers. In response, the remaining health-facility-level packages of the MHCP are separated over two cadres of primary healthcare workers: prescribers (the group that is mandated to prescribe medicine) and non-prescribers (healthcare providers that are not mandated to prescribe medicines). Prescribers are thus trained to deliver pharmacological treatment when indicated, especially for people with psychoses, epilepsy and severe depression (as well as moderate depression if psychosocial support does not result in improvements) (package 2.6 in Table 1). Ongoing supervision for the prescribers is provided by a visiting psychiatrist. The non-prescribing health staff are responsible for providing brief focused manualised problem-oriented psychosocial support (package 2.5 in Table 1). For depression the health workers are trained to deliver a brief intervention based on behaviour activation principles derived from the Healthy Activity Program,^18^ and for alcohol use disorders a brief intervention based on motivational interviewing derived from the Counselling for Alcohol Problems intervention.^19^ In addition, a separate group of health workers are capacitated to deliver a cognitive–behavioural therapy-based intervention specifically for maternal depression, i.e. the Thinking Healthy Program – an intervention with proven effectiveness in a similar setting.^20^ Supervision for the psychosocial support package is provided by a TPO Nepal counsellor.

Third, to ensure tertiary care for people with severe and persistent mental disorders that cannot be treated within primary healthcare facilities, referral mechanisms are established (package 2.1 in Table 1). This is done through training of the primary healthcare workers on adequate referral pathways and in the use of a simple screen for suicidal ideation and self-harm.

Fourth, the final package is geared towards ensuring continuing care to patients that have entered the MHCP (package 2.7 in Table 1). Follow-up is stimulated through active monitoring of patients' progress and need for continued care. Home visits (i.e. home-based care) by community health workers (i.e. female community health volunteers) focuses on monitoring treatment adherence and need for follow-up consultation.

### Community care delivery platform

The aims of the community-level care packages are to improve access to care, and to contribute to improved outcomes and social inclusion for people with mental health problems. The interplay between the health facility and community-level packages is central to the MHCP, one cannot function without the other to cover both demand-side and supply-side issues related to integrated mental healthcare. The following packages are included.

Community sensitisation and stigma reduction (package 3.1 in Table 1) aims to increase mental health literacy among the community at large, including knowledge on the (now) available services. Further, it aims to reduce stigmatising attitudes and discrimination towards people with mental health problems. Similar to the anti-stigma intervention for primary healthcare workers described above, at the community level interactions are organised between different stakeholder groups (i.e. media, teachers) and patients along with family members, aiming to facilitate social engagement, skills associated with stigma reduction and developing community action plans. Also, large group community meetings are convened for awareness raising, supported by information leaflets.

A barrier raised by the formative study is the low demand for mental healthcare. To pre-empt this, we developed the community informant detection tool (CIDT) (online Fig. DS3), which is a procedure for proactive case-finding of people with probable mental health problems and subsequently promotes help-seeking (package 3.2 in Table 1). The CIDT consists of contextualised vignettes and associated pictures to facilitate recognition by lay people. The rationale behind the strategy is that briefly trained community members (i.e. female community health volunteers and mothers groups) that are intimately familiar with the community, are especially well placed and capable of identifying people in need of care. Preliminary research into the accuracy of the CIDT confirms this.^21^

To complement the brief focused psychosocial support provided by the primary healthcare workers, a cadre of community-based counsellors is introduced to provide the complete psychological treatments following established protocols. Primary healthcare workers can therefore refer cases to the community counsellors if more advanced psychosocial support is indicated (package 3.3 in Table 1). The community are trained in generic counselling (including emotional support and problem-solving skills), as well as different protocolised interventions: the behaviour-activation-based Healthy Activity Program for people with depression,^18^ motivational-interviewing-based Counselling for Alcohol Problems for people with alcohol use disorders,^19^ the Thinking Healthy Program for maternal depression^20^ and family counselling for people with psychoses or epilepsy. The latter component is a response to recommendations generated from the formative research, which highlights the need to work systematically with the families of people with severe conditions to improve acceptability of, and adherence to, treatment. Ongoing supervision of the community counsellors is provided by a psychologist.

The final care package, user group mobilisation, aims to connect people with mental illness to health facilities and to promote peer support (package 3.4 in Table 1). This is done through peer support groups, a regular group session for patients, initially facilitated by counsellors.^22^ After formation, the peer support groups are geared towards establishing social support and facilitating linkages to existing community resources (such as income generation opportunities) for the group.

Supervision is an integral part in the MHCP, especially as previous endeavours have demonstrated that one-off training courses are insufficient to successfully capacitate health workers.^23^ Ongoing supervision is offered to all staff involved in service provision. The supervision structure follows a cascading approach, and ranges from case conferences with a psychiatrist, clinical spot checks by a psychiatric nurse, managerial supervision meetings by district health administration and peer supervision for psychosocial care by the community counsellors, who in turn receive supervision from a psychologist (Table 2). Supervision will be the main mechanism for quality assurance, complemented by two tools. A register will be used to record all diagnosed patients and the subsequent services provided. We will also use the Enhancing Assessment of Common Therapeutic Factors (ENACT) rating scale, a tool to assess health workers' competence in psychological treatments and other mental health services.^24^

**Fig. 1:**
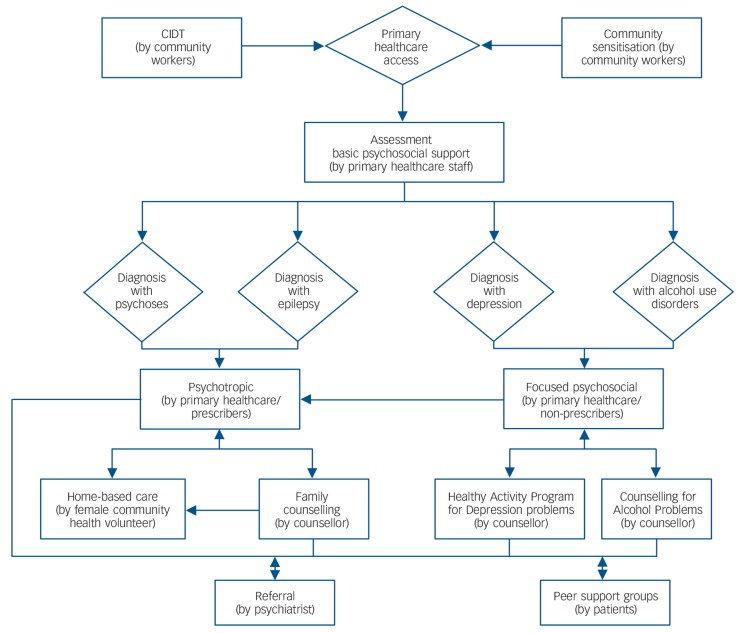
Service delivery framework. CIDT, community informant detection tool.

In practice the above-mentioned separate packages form an integrated service delivery framework wherein people can flow between the different parts and levels of the system of care, depending on need (Fig. 1). Given the scarce mental health resources, the entire MHCP is built on the notion of task-sharing, with non-specialists taking on care functions otherwise done by mental health specialists. A number of capacity-building trajectories are required to achieve this. For the health workers, a stepped training approach ensures that all health facility staff (service providers and administrative) receive an introductory course, with added levels of training for specific functions for different cadres of healthcare workers. Similarly, the community counsellors also follow a staged training structure, with a basic training course focusing on core and generic therapeutic competencies, subsequently combined with the protocolised psychological treatments for specific mental health problems. All training courses are competency- and skills-focused and are followed up with brief refresher courses and supervision.

### Pilot-testing results

During the 11-month pilot period, the packages of the MHCP were initiated in the two health facilities and surrounding communities. The implementation steps included the training of different cadres of health workers in mental health awareness and stigma reduction, assessment and treatment (both pharmacological and psychosocial). At the same time, the community health workers were trained and mobilised for conducting sensitisation programmes and proactive case-finding, and community counsellors commenced psychological treatments. Supply and management of medicines was organised and supervision initiated. The recovery packages (i.e. continuing care and user group mobilisation), the health organisation package (i.e. policy engagement) and the stigma reduction components were not included in the pilot. Table 3 presents an overview of all the people (*n* = 135) who were diagnosed by trained health workers and subsequently received treatment during the pilot period. The most frequent diagnosis conferred by the health workers trained in the mhGAP assessment and treatment guide (i.e. prescribers) was depression (37%), followed by psychosis (24%). Dropping out (21%, i.e. attending only one session and not returning for follow-up within the following 3 months) was most common among the alcohol use disorders group (36%, *n* = 10), followed by epilepsy, psychoses (both 25%, *n* = 7) and depression (14%, *n* = 4). The remaining 79% of the sample received at least one or more of the mental healthcare components (i.e. basic, focused or advanced psychosocial care or pharmacological treatment), with an average of 4.24 (s.d. = 3.35, median 4.0) health facility visits in the reporting period.

**Table 3:** Patient demographics and service utilisation (*n* = 135)

	Total, *n* (%)
Female	79 (58.5)

Age	
Below 18	6 (4.4)
18–24	15 (11.1)
25–59	74 (54.8)
Above 60	13 (9.6)
Not recorded	27 (20.0)

Ethnicity	
Brahmin/Chettri	62 (45.9)
Tharu	43 (31.9)
Dalit	12 (8.9)
Other	18 (13.3)

Diagnoses	
Psychosis	32 (23.7)
Depression	50 (37.0)
Epilepsy	20 (14.8)
Alcohol use disorders	16 (11.9)
Other	17 (12.6)

Service utilisation	
One visit (no follow-up)	28 (20.7)
Two visits	13 (9.6)
Regular visits (>2); at monthly follow-up	74 (54.8)
Referred to other services	17 (12.6)
Not recorded	3 (2.2)

*Type of services*	
Medicines (by primary healthcare worker)	82 (60.7)
Basic psychosocial support (by primary healthcare worker)	52 (38.5)
Emotional support	22 (16.3)
Psychoeducation	52 (38.5)
Stress management	9 (6.7)
Focused psychosocial support (by primary healthcare worker)	78 (57.8)
Behaviour activation	54 (40.0)
Motivational interviewing	23 (17.0)
Family support	10 (7.4)
Advanced psychosocial support (by community counsellor)	35 (26.0)
Not recorded	30 (22.2)

Next, we reviewed service utilisation data to assess the allocation of treatment to different patient groups. Among people with depression (*n* = 50), 64% received focused psychosocial support from primary healthcare workers, 24% from a counsellor and 40% were prescribed psychotropic medicines. All 32 patients with psychosis were prescribed medicines and 18% also received family counselling. Similarly, for people with epilepsy (*n* = 20), 95% received medication and 15% family counselling. Finally, for people with alcohol use disorders (*n* = 16), 44% received focused psychosocial support from primary healthcare workers, 31% from counsellors and 63% were prescribed psychotropic medicines. The 35 patients that received support from the community counsellors attended an average of 5.5 sessions at the time of data collection, with a 37% termination rate.

Among the group of patients that were asked to complete a routine evaluation questionnaire (*n* = 45, i.e. individuals who continued care after the first session), the majority indicated that they were somewhat or very satisfied on most of the indicators (Table 4). Satisfaction was especially high on perceived improvements and the time provided by the service provider. Patients report a high rate of endorsement for improvement after treatment (80% score somewhat or completely). A large majority (87%, *n* = 39) reported that they would seek help again for their problems from this health facility or counsellor in the future. On three indicators a subgroup of patients (between 15% (*n* = 7) and 22% (*n* = 10)) express clear dissatisfaction with respect to the perceived relevance and appropriateness of care and overall satisfaction. The main reasons for dissatisfaction were related to the unavailability of the medicines as prescribed by the specialists. Also, the level of satisfaction with privacy was clearly lower than other indicators, with almost 64% (*n* = 29) only a little satisfied or less. The lack of private rooms in most of the health facilities means that many consultations are done in the space that also serves as the waiting area.

**Table 4:** Routine evaluation data

	*n* (%)				
	Not at all	Hardly	A Little	Somewhat	Completely
Patients, *n* = 45^a^					
To what degree do you feel that this was a sufficient amount of time?	2 (4.4)	2 (4.4)	5 (11.1)	26 (57.8)	10 (22.2)
To what degree was the treatment as you expected?	8 (17.8)	5 (11.1)	6 (13.3)	21 (46.7)	5 (11.1)
To what degree do you feel the treatment you received was appropriate for your complaint?	7 (15.6)	3 (6.7)	8 (17.8)	18 (40.0)	9 (20.0)
To what extent do you feel that the primary healthcare worker was sensitive to your personal needs?	3 (6.8)	3 (6.8)	5 (11.4)	31 (68.2)	3 (6.8)
To what degree did you feel comfortable with levels of privacy during your treatment?	2 (4.4)	1 (2.2)	26 (57.8)	15 (33.3)	1 (2.2)
Have you experienced any improvements since your first visit?	3 (6.7)	1 (2.2)	5 (11.1)	25 (55.6)	11 (24.4)
Overall satisfaction with the service for this problem	10 (22.2)	2 (4.4)	5 (11.1)	25 (55.6)	3 (6.7)

Health workers, *n* = 11					
Your overall level of distress in providing this treatment?	4 (36.6)	0	3 (27.3)	4 (36.4)	0
Your satisfaction with overall outcome of sessions?	0	1 (9.1)	2 (18.2)	6 (54.6)	2 (18.2)

a. Includes completed evaluation questionnaires of individuals who have received regular services; not all rows add up to 100% as some responses were left blank.

The 11 health workers who were asked to complete routine service provider evaluation forms shared that providing mental health services has not been easy for them. About a third of them (36%, *n* = 4) reported being ‘somewhat distressed’ in providing mental health treatment (Table 4). The health workers perceive the additional time spent on patients as burdensome. Still, they were satisfied with the outcomes of the provided care (73% (*n* = 8) reported being somewhat or very satisfied). In addition, during the formative study, health workers expressed that a clear mandate is required for them to be able to perform mental health tasks.

We also followed-up with a group of people who did not return to treatment after the first session (*n* = 28) to understand their reasons for dropping out. This was done because treatment follow-up was one of the largest challenges encountered during the piloting phase, especially for people with alcohol use disorders. The most frequently mentioned reasons included side-effects of medication (among people with antipsychotic and anti-epileptic medicines) (*n* = 6), time constraints (*n* = 5), unavailability of prescribed medicine (*n* = 5), belief that no treatment is needed because the problem can be solved by themselves (*n* = 4), has seen sufficient improvement (*n* = 4), claims not to have any (mental health) problems at all (*n* = 3) and distance (*n* = 1).

## Discussion

### Main findings

In addition to demonstrating the need for, and efficacy of, mental health treatments in LMIC, increasingly there is a need for a realistic service delivery model that demonstrates how the treatment gap can be overcome. This is especially the case for fragile states with a weak care infrastructure.^25^ Several authors have described the challenges and opportunities related to the integration of mental health into primary healthcare in post-conflict settings,^6,26,27^ yet content descriptions of the care packages or implementation data are scarce. The results of the pilot testing of the MHCP developed in Nepal provide preliminary support for its applicability and adequacy. First, the allocation of treatments for different patients (i.e. client flow through the care system for people with psychoses, depression, epilepsy or alcohol use disorders) was largely as intended, many of whom received a variety of care packages. For any care system to work suitably it is important that individuals utilise different parts of the system, according to individual needs.^28^ Second, perceived satisfaction with utilised (and provided) services was quite good overall, with high satisfaction scores on most indicators, especially on important indicators like perceived outcome and time spent with service provider. Satisfaction has been associated with beneficial outcome.^29,30^ This type of data is valuable in assessing how the care package is functioning, yet is often missing for mental health services in low-income settings.^31^

### Fine-tuning

Development of the MHCP followed a multistepped process. A core structure was designed based on a conceptual model and a ToC framework. A first set of adaptations were made based on the formative research.^14^ Finally, as a result of pilot testing (provisional implementation of care package, routine monitoring and evaluation data of service utilisation), another set of alterations have been applied to fine-tune the content and delivery mechanisms, resulting in the current version of the MHCP that will be rolled out in the district.

First, the continuing care package in the care plan required a more proactive approach, through assertive outreach work, to target high treatment discontinuation. Consequently, more active involvement of family members is incorporated through adding home visits to the plan, and emphasising the family support intervention by the community counsellors.

Second, the community counsellors have been largely unable to terminate treatments with any of the patients who started it. Although the patients were highly satisfied with the counselling services, non-termination will obviously become a capacity problem when the plan will be rolled out to a larger catchment area. Hence, the interventions provided by the community counsellors have been more strictly manualised, with accompanying session-by-session ‘prompt-sheets’ (concise notes on session content used for quick reference) to assist counsellors in remaining on track.

Third, health workers expressed both being content (high satisfaction scores on perceived effect of the delivered care) and burdened by the ‘supplemented tasks’ (high perceived distress). It was apparent from the formative study and the pilot period that a clear task division is needed among the health workers in order to maximise the little available time. To address the distress, we have further adjusted this division by having all focused psychosocial support offered by one cadre (non-prescribers), relieving the prescribers from providing this care. Moreover, the scope of the focused psychosocial support intervention for the health workers has been reduced, now focusing on the only key ingredients that can be delivered in few and brief sessions (i.e. brief focused psychosocial support: approximately three sessions, with 15 min per session). As opposed to the brief interventions by health workers, the community counsellors will deliver the complete treatment protocols for the psychological treatments. Patients in need of advanced support can thus be referred from the primary healthcare workers to the community counsellors.

Fourth, the need for a clear mandate, expressed through recognised certification, is an important aspect of the acceptability of the care package by health workers. We acted on this need by having all training completion certificates co-issued by the Ministry of Health and Population, and the training curriculum recognised by the National Health Training Centre, the official body regulating health worker education. In addition, practical tools (such as a simplified flow chart and pocket booklets) were developed to support, promote and legitimise the interventions.

Finally, discontent on appropriateness of treatment and privacy exists among a significant subgroup of patients. Unavailability of some regular medication was one of the main reasons for the low score on appropriateness. The pharmacological treatment package now includes medications that are commonly prescribed by specialists in the district headquarters. Future efforts still need to address the dissatisfaction with privacy – a challenging endeavour given the lack of separate rooms in most facilities.

Nepal has seen several initiatives towards the integration of mental health into primary healthcare,^7,8^ but these have not resulted in a replicable plan. Although the MHCPs across the PRIME sites have a similar structure,^32^ there are a number of aspects that are specific to Nepal. We propose a levelled capacity-building approach among the health workers, thereby aiming to include all health facility staff albeit at different skill levels. This includes distinct psychosocial support skills and interventions. Also, the plan involves a new cadre of community counsellors that serve to bridge the gap between community-based care and facility-based care. This service delivery agent can safeguard and bolster the psychosocial care function within the overall care system. It should be noted that this is a new position within the Nepal health system, thus requiring significant additional resources. At the same time, extensive experience in community psychosocial interventions by TPO Nepal should ensure the feasibility of including this care package within the overall MHCP, at least initially.^9^ We are currently evaluating the added benefit of community counsellors to the overall care plan, using a randomised controlled trial design. A strategy has been developed to increase community detection, this is the development and use of a proactive case-finding strategy, the CIDT, to identify people with probable mental health problems using vignettes that are context sensitive.^21^ Overall, the plan is congruent with the recent paradigm shift towards collaborative care models, which involves transferring service delivery tasks to community and primary health workers and mental health professionals taking up training, supervision and referral roles,^33,34^ incorporating non-health sector cadres and interventions.

### Challenges and current limitations

There are a number of challenges and limitations to be mentioned. During the pilot testing of the plan, not all packages were implemented. The health organisation package, peer support groups, the psychosocial intervention for maternal depression and targeted stigma reduction interventions have not yet been included, for reasons mentioned. The presented data is the information that was available through routine monitoring and evaluation (following a system put in place by the PRIME programme) and as recorded by the service providers, and therefore does not address issues of quality, fidelity, intensity and outcome of services or accuracy of diagnosis. The adequacy of prescriptions of psychotropic medications requires further investigation, especially for people with depression and alcohol use disorders. The high rate of prescription for alcohol use disorders requires attention, as it might indicate irrational drug use. Quality assurance mechanisms will be systematically integrated in supervision. Although supervision does involve reviewing accuracy of diagnosis and subsequent treatments, this was not systematically documented as part of this study, mainly because a large-scale study evaluating the accuracy of primary healthcare workers diagnosis is currently under way.^35^ Neither does it address the accuracy of the registration of provided services by the health workers. From supervision sessions it appears that health workers are reporting delivery of the focused psychosocial care components even if applied partly, which explains why focused psychosocial support is reported more frequently than basic psychosocial support components that tend to be under-registered as it consists of less clearly demarcated tasks. The issue of record-keeping will be addressed in continued capacity-building efforts. This pilot study does not assess the feasibility of health system requirements necessary for the implementation of the MHCP, in particular issues of required human resources (estimated 14.3 full-time equivalent staff per 100 000), procurement and supply chain management of medicines, and related costs, are addressed in a related study.^36^

As Chitwan is a more developed district compared with many districts in Nepal, further research will need to demonstrate the transferability of the MHCP to more remote areas of the country with less existing care infrastructure. Currently, another project is underway to pilot test the approach in such an area (Pyuthan district). Government commitment and investments into mental health services beyond the programme period and area is unsure. Still, there are several policy developments underway that promote scaling up of mental healthcare (such as a new health act currently being drafted and a revised essential drug list to include additional psychotropic medicines), some explicitly supporting the use of counsellors.

In conclusion, in this paper we have presented the content of a mental healthcare delivery system, as well as initial implementation data supporting its real-life applicability. It has yielded further improvements to increase feasibility and acceptability (i.e. strategies to reduce drop-out, dissatisfaction and service provider burden). The developed MHCP will serve as a template for roll-out to the entire district and as the ‘final protocol’ for the studies evaluating the impact of the plan on improved patient outcomes, detection and treatment coverage.^35^ The overall aim is for the MHCP to serve as a tool to aid the Government of Nepal to implement the inactive policy towards the integration of mental health into primary healthcare.
